# The Therapeutic Potential of Celastrol in Central Nervous System Disorders: Highlights from In Vitro and In Vivo Approaches

**DOI:** 10.3390/molecules26154700

**Published:** 2021-08-03

**Authors:** Stefania Schiavone, Maria Grazia Morgese, Paolo Tucci, Luigia Trabace

**Affiliations:** Department of Clinical and Experimental Medicine, University of Foggia, Via Napoli 20, 71122 Foggia, Italy; mariagrazia.morgese@unifg.it (M.G.M.); paolo.tucci@unifg.it (P.T.); luigia.trabace@unifg.it (L.T.)

**Keywords:** celastrol, neurodegenerative diseases, neuropsychiatric disorders, animal models, in vitro

## Abstract

Celastrol, the most abundant compound derived from the root of *Tripterygium wilfordii,* largely used in traditional Chinese medicine, has shown preclinical and clinical efficacy for a broad range of disorders, acting via numerous mechanisms, including the induction of the expression of several neuroprotective factors, the inhibition of cellular apoptosis, and the decrease of reactive oxygen species (ROS). Given the crucial implication of these pathways in the pathogenesis of Central Nervous System disorders, both in vitro and in vivo studies have focused their attention on the possible use of this compound in these diseases. However, although most of the available studies have reported significant neuroprotective effects of celastrol in cellular and animal models of these pathological conditions, some of these data could not be replicated. This review aims to discuss current in vitro and in vivo lines of evidence on the therapeutic potential of celastrol in neurodegenerative diseases, including Alzheimer’s and Parkinson’s diseases, amyotrophic lateral sclerosis, Huntington’s disease, multiple sclerosis, and cadmium-induced neurodegeneration, as well as in psychiatric disorders, such as psychosis and depression. In vitro and in vivo studies focused on celastrol effects in cerebral ischemia, ischemic stroke, traumatic brain injury, and epilepsy are also described.

## 1. Introduction

**Figure 1:**
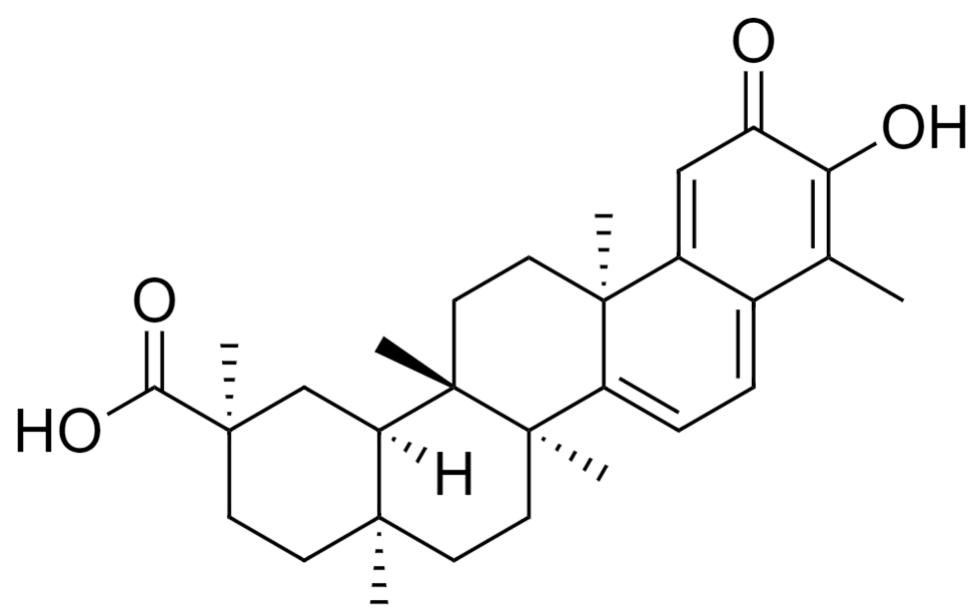
Celastrol chemical structure (drawn by the ChemDraw Software, version 12).

Celastrol (3-hydroxy-9β,13α-dimethyl-2-oxo-24,25,26-trinoroleana-1(10),3,5,7-tetraen-29-oic acid) (Figure 1), is a natural compound derived from different plant species of Celastracae family, such as *Celastrus paniculatus*, *Celastrus scandens*, *Celastrus strigillosus*, *Catha cassinoides*, *Kokoona ochrasia*, *Kokoona zeylanica*, *Maytenus umbellata*, *Mortonia greggii*, *Mortonia palmeri*, *Orthosphenia mexicana*, *Salacia reticulata* var. ß-diandra, and *Tripterygium wilfordii* [1], also called “Thunder God Vine”. Celastrol quantities in these plants are low; in particular, in Tripterygium root extracts, its content is about 1% of dry biomass [2]. Moreover, the harvest of the plant is subject to restriction and its chemical synthesis is difficult and expensive [3]. Celastrol has been used in traditional Chinese medicine for a broad range of disorders [4,5,6,7]. 

Indeed, this compound has been shown to have clinical efficacy in several diseases and pathological conditions such as rheumatoid arthritis [8], kidney transplantation [9], neoplastic disorders, and infertility [10]. Despite its different biological properties, still limited evidence are available on celastrol possible cellular targets [6]. Concerning its mode of action, it has been shown to conjugate addition by cysteine residues [11,12,13] and to impact on different signaling pathways, such as the ones related to Hsp90 [14], Cdc37 [15], p23 [16], and IKKβ [17]. 

Preclinical lines of evidence have reported that celastrol also has beneficial effects in different pathological conditions characterized by an overproduction of reactive oxygen species (ROS) [18]. In this regard, it has been widely reported that ROS act as key mediators of brain injury and, therefore, are crucially involved in the pathogenesis of Central Nervous System (CNS) disorders [19]. Celastrol has been described to directly inhibit the ROS producer NADPH oxidase NOX enzymes, with an increased potency against NOX1 and NOX2 isoforms, via a novel mode of action, i.e., the inhibition of the functional association between the cytosolic subunits and the membrane flavocytochrome [18]. NOX enzymes have been described as one of the major source of ROS in the CNS, being crucially implicated in the development of its pathological conditions [20]. Thus, increasing interest has raised so far regarding possible beneficial effects of celastrol in CNS diseases. 

In this review, we discuss current knowledge on the therapeutic potential of celastrol in different pathological conditions of the CNS, i.e., neurodegenerative diseases (Alzheimer’s disease, Parkinson’s disease, amyotrophic lateral sclerosis, Huntington’s disease, multiple sclerosis, and cadmium-induced neurodegeneration), psychiatric disorders, such as psychosis and depression, as well as cerebral ischemia and ischemic stroke, traumatic brain injury, and epilepsy. To this aim, we summarize lines of evidence obtained on in vitro and animal models of these disorders. A critical conclusion on still lacking aspects of this field of research is also provided. 

## 2. Literature Search Methods

### 2.1. Peer-Reviewed Publications

In this review, we included peer-reviewed papers, published in PubMed from 1 January 2000 to 31 May 2021. The combinations of keywords used for literature search are available in the Supplementary Material. Only peer-reviewed original research articles and reviews written in English were assessed for evaluation and inclusion in the present manuscript. Publications resulting in PubMed that did not address in any of their sections celastrol use were excluded from the evaluation. No publications focused on the therapeutic potential of celastrol in anxiety, bipolar disorders, hypomania and mania, autism and autism spectrum disorders, as well as sleep disorders, were found by using the above-mentioned keyword combinations.

### 2.2. International Patents 

We searched for international patents disclosing in vitro and in vivo findings on the use of celastrol in CNS disorders in “The World Intellectual Property Organization (WIPO)-Patent Scope” website (https://patentscope.wipo.int/search/en/search.jsf (accessed on 25 May 2021)), using, for the refine search, the following terms: “celastrol and CNS”. Five patents resulted from this searching. However, only two of them disclosed findings related to CNS pathology (namely, neurodegenerative disorders and epilepsy) and were, therefore, considered for inclusion in this review. 

The Introduction section and the opening statements of the different sections of the manuscript also enclosed some additional references which were cited for the background descriptions and which were, therefore, listed in the final reference list. 

## 3. The Therapeutic Potential of Celastrol in Neurodegenerative Disorders

Neurodegenerative diseases, characterized by protein aggregates accumulation, are also known as “protein misfolding disorders” [21]. The Heatshock proteins (Hsps) have been reported to play a crucial role in the defense process against the formation of these protein aggregates [22]. It has been reported that celastrol is able to induce the expression of neuroprotective Hsps in both rodent and human differentiated neuronal cell lines [23,24]. However, studies on human cells showed that administration of this compound determined greater neuroprotective effects than in rodents [25,26,27]. This mainly occurs via a more significant induction of the Hsp70B expression, resulting in the inhibition of NF-κB-mediated inflammatory pathways [28,29] and in the suppression of the immune response [30]. 

Peer-reviewed publications reporting in vitro and in vivo lines of evidence on the effects of celastrol in Alzheimer’s and Parkinson’s disease, as well as Amyotrophic lateral sclerosis and Huntington’s disease, are reported in Table 1. 

In 2004, in vitro evidence related to the use of celastrol and its derivatives in neurodegenerative disorders were also disclosed in an international patent (publication number: US 2004/0220267 A1, Inventor: J. P. Devlin, Bridgewater, CT (US)). In particular, the inventor reported that dihydro-derivatives of celastrol could significantly increase the Hsp70 generation in cells. Moreover, dihydrocelastrol and dihydrocelastrol diacetate were also reported to inhibit protein aggregation in the “in vitro Huntington Aggregation assay”.

Multiple sclerosis (MS) is a neurodegenerative demyelinating autoimmune disorder, affecting the brain and the spinal cord for which the available immunomodulatory or anti-inflammatory therapies have shown limited beneficial effects and significant adverse reactions. The impact of celastrol on the pathological mechanisms underlying MS onset and progression has been reported by a limited number of works performed on a rodent model of the disease, i.e. the experimental autoimmune encephalomyelitis (EAE). In this context, Abdin and Hasby investigated the possible impact of celastrol treatment on the release of Th1/Th2 cytokines, on TLR2 expression, as well as on the number of CD3+T-lymphocytes in EAE rats, showing that administration of this compound to EAE rats lead to a significant improvement of their clinical score and inhibited the disease relapse, shifting the cytokines profile from Th1 to Th2 production, decreasing the expression of NF-κB and TLR2, as well as reducing the nitrite amount and the CD3+ T-lymphocytic count [43]. In an elegant work where the EAE model in female mice was used to elucidate celastrol impact in MS development, a significant attenuation of EAE symptoms was obtained after a 4 day-intraperitoneal (i.p.) treatment with this compound. This was associated to an inhibition of the immune response in peripheral lymph nodes, in terms of IL-17 production decrease, reduction of the CD4+IL-17+ cells and diminished lymphocytes proliferation, as well as to the suppression of the mRNA of specific genes, also including RORγt and STAT3. In addition, in this same work, celastrol was shown to dramatically inhibit NF-κB activation and suppress p65 phosphorylation in bone-marrow derived dendritic cells, where an abrogation of the production of other inflammation mediators, such as IL-1β, IL-6, and TNF-α was also found. This was observed also in splenic dendritic cells, whereas the process of the intrinsic differentiation of T-cells could not be suppressed by celastrol. When naive T cells were co-cultured with bone marrow or splenic-derived dendritic cells, in the presence of LPS but in the absence of any exogenous cytokines, and treated with celastrol during 4 h, a suppression of both IL-17 mRNA and protein levels was detected. The same was observed when dendritic cells derived from the spleen of EAE mice were co-cultured with naive T cells. Finally, the Authors reported the absence of celastrol effects on CNS T-cell invasion, rather on the CNS infiltration by this cell type [44]. In line with these findings, Yang and Co-workers described the beneficial effects of celastrol treatment (during 13 days) in EAE rat, where, despite the lack of effects on the disease onset at both low and high doses, this compound was able to ameliorate the severity of the neurologic symptoms. This was associated to the suppression of EAE-induced histopathological alterations and demyelination in the spinal cord, where the production of pro-inflammatory cytokines, including IL-17 and IFN-γ, was increased, whereas levels of IL-4, playing a crucial role in the recovery of the disease, was enhanced. When evaluating celastrol effects on the optic nerve of EAE rats, inhibited cytokine production and microgliosis, together with suppression of iNOS and of NF-κB activation, as well as reduced retinic apoptotic phenomena, were described following administration of this compound [45]. In a recent publication, celastrol was reported to regulate the expression of the encoding serum/glucocorticoid regulated kinase 1 (SGK1) gene, crucially involved in the process of Th17/Treg differentiation, via a MAPK pathway-mediated molecular mechanism and to provide neuroprotection against EAE mice by promoting BDNF expression [46].

## 4. The Therapeutic Potential of Celastrol in Neurodegenerative Disorders Induced by Cadmium

Toxic environmental contaminants, especially cadmium, may induce neurodegenerative processes in the CNS via different mechanisms of action [47,48]. Chen and co-workers reported that celastrol administration was able to significantly decrease the viability reduction induced by cadmium in neuronal cells, also attenuating other detrimental effects induced by this toxic metal, such as nuclear morphological alterations, in terms of fragmentation and condensation, as well as increased caspase-3 activation. Furthermore, celastrol was found to protect neuronal cells from death by inhibiting the phosphorylation process of the N-terminal of the c-Jun kinase and preventing the activation of Akt/mTOR signaling [49]. Cadmium has been also described to induce the expression of the NADPH oxidase NOX2 together with its regulatory proteins p22phox, p40phox, p47phox, p67phox, and Rac1 and to enhance ROS production in different cell lines, including PC12 cells and primary neurons [50]. Celastrol prevented these cadmium-induced effects in the above-mentioned cell lines and potentiated the neuroprotective effects of ROS scavengers or antioxidant/NOX inhibitor compounds, such as N-acetyl-l-cysteine and apocynin respectively, through the blocking of the production of NOX2-derived free radicals [51]. Furthermore, attenuation of intracellular-free calcium [Ca2+ ]i amount and of consequent neuronal cell apoptosis, via the inhibition of the Akt-mediated mTOR pathway and the hinding of [Ca2+ ]i -mediated CaMKII phosphorylation, was reported following administration of celastrol [52]. By using PC12, SH-SY5Y cells and primary murine neurons, Zhang and collaborators showed that alterations in cell viability, apoptosis, production of free radicals, and activation of AMPK/mTOR pathway induced by cadmiun were attenuated by celastrol administration. In particular, this molecule was found to suppress both cadmium-induced mTOR activation and ROS production by mitochondria [53]. 

## 5. The Therapeutic Potential of Celastrol in Neuropsychiatric Disorders

As regard to neuropsychiatric disorders, we have previously demonstrated that oxidative stress is a predisposing factor to psychotic like behavior in rodents [54,55]. Furthermore, we also evidenced that early post-natal insult, represented by ketamine administration in post-natal days 7–9–11, led to psychotic-like behaviors accompanied by oxidative disequilibrium and neurochemical impairment in prefrontal cortex and cerebellum of treated mice at adulthood [56,57]. In addition, we demonstrated that early celastrol administration counterbalanced the increase in cortical ROS production, in the enhanced lipid peroxidation, and in inflammation induced by ketamine administration. Thus, our findings indicated that celastrol might represent a useful adjuvant therapy for psychotic-like conditions through the enhancing of antioxidant defense along with inhibition of neuroinflammatory pathway release. 

In regard to depressive-like phenotype induced in rodents, celastrol administration was found to be effective in preclinical paradigm of depressive-like phenotype associated with other pathological conditions, such as obesity. In particular, it was reported that in a mouse model of diet-induced obesity in which the injection of purified TNF-α protein in basolateral amygdala induced depressive behaviors, celastrol reverted the increased immobility in the forced swimming test and tail suspension test. In this model, celastrol accelerated the degradation of heterogeneous nuclear ribonucleoprotein A1 (HnRNPA1), a mediator of the proteasome-dependent degradation of IKBα, a strong activator of NF-κB, thus leading to enhanced transcription of TNF-α [58]. Furthermore, celastrol was found to be effective in another animal model of depression, such as the winter depression-like behavior reproduced in Medaka fish. In this model, seasonal changes in the nuclear factor erythroid-related factor 2 (NRF2) antioxidant pathway were reported and celastrol was found to be able to activate NRF2and to reverse such behavioral phenotype [59]. In particular, the anti-inflammatory properties of celastrol were demonstrated through the activation of the NRF2 antioxidant pathway and in turn NRF2 deletion corresponds to a depressive like phenotype in transgenic mice [60]. Furthermore, celastrol might result useful also in other type of disorders comorbid with depression such as Alzheimer’s disease or in animal model of Aβ-induced depressive like phenotype [61]. Indeed, antioxidant treatments were found to positively reduce depressive behavior in this animal model [62,63]. In good agreement, celastrol was found to be able to affect beta-amyloid production in transgenic Alzheimer’s disease models interacting with BACE-1 through a NF-kB dependent mechanism [32]. In this regard, drugs able to lower NF-kB expression in model of Aβ-induced toxicity were also associated with improved depressive-like behavioral outcomes [64], thus further studies are warranted in order to endorse the proposed anti-depressant effect of celastrol also in this animal model.

## 6. The Therapeutic Potential of Celastrol in Cerebral Ischemia, Ischemic Stroke, and Traumatic Brain Injury 

Increased oxidative stress and inflammatory insult have been reported to act as key players in the pathogenesis of cerebral ischemia and ischemic stroke. Thus, given its antioxidant and anti-inflammatory properties, celastrol has been tested for potential beneficial effects both in vitro and in animal models of these CNS disorders (Table 2). 

## 7. The Therapeutic Potential of Celastrol in Epilepsy

Among CNS disorders, epilepsy is undoubtedly characterized by a high number of pharmacological opportunities, but, at the same time, by a significant rate of drug resistance [70]. Therefore, most of the pharmacological research in the field is focused on the possibility to identify novel molecular mechanisms crucially involved in the pathogenesis of the disease in order to develop innovative therapeutic approaches both to overcome the pharmacologic resistance and to prevent seizure recurrency. In this context, a recent publication by Malkov and co-Authors identified the NMDA-mediated activation of the NADPH oxidase NOX enzymes, with consequent H_2_O_2_ rapid release, as the primary trigger of epileptic seizures in animal models of this neurological disorder. By using both an in vitro and in vivo approaches, they reported that NOX blockade by celastrol administration was able to prevent H_2_O_2_ release and the consequent seizure-like events in hippocampal slices, as well as the kainic acid-induced seizures in rats [71]. 

The role of immunity and inflammation in epileptogenesis has been also widely described [72,73]. In this context, by using the multiple-hit rat model, Shandra and co-writers reported preclinical evidence about the rapid beneficial effects of celastrol administration on infantile spasms via NF-kB inhibition [74]. On 8 February 2018, the Vanderbilt University, Kirkland Hall, Nashville, Tennessee (US), published a patent (International Publication Number: WO 2018/026810 Al, International Patent Classification: C07D 487/22 (2006.01), International Application Number: PCT/US20 17/044893, inventor: KANG, Jing-Qiong) in which it was stated that “*celastrol possess acceptable to favorable properties for a CNS drug*”. The patent disclosed the use of this compound in mouse models of epilepsy, celastrol ability in attenuating seizure severity, as well as in improving seizure-associated dysfunctions in learning and memory, and the biochemical pathways underlying celastrol effects in cells expressing the mutant GABAA receptor subunits. In particular, the patent provided preclinical evidence about the beneficial effects of chronic celastrol i.p. or oral administration in decreasing seizure activity, via different mechanisms, including GABAergic neurotransmission enhancement, protein homeostasis restoration and increase of both the surface and total GABAA receptor subunits. These findings have been obtained by using two rodent models of the Dravet syndrome, a severe epileptic encephalopathy occurring in early childhood, implicating a high rate of morbidity and mortality [75], i.e., the Gabrg2+^/Q390X^ and Scn1a^+/–^ mouse models. More specifically, in Gabrg2+^/Q390X^ mice, celastrol could decrease the frequency of EEGs spike-wave-discharges in a dose-dependent manner, having the dose of 0.3 mg/kg i.p. daily for 14 days good efficacy and tolerability, whereas for mice treated with doses between 3 and 6 mg/kg i.p. some important side effects including lethargy, fur alterations and weight loss were detected. Celastrol was also found to remove the accumulation of the mutant protein derived from the GABRG2 (Q390X) mutation and associated with an exacerbation of the disease, this resulting finally in an improvement of the pathology outcome. In addition, in this animal model, celastrol administration has been reported to exert beneficial effects on the reduction of the current amplitude of the receptor channel caused by the mutation. Data obtained on GABAergic mlPSCs from pyramidal neurons of the cortical layer VI derived from Gabrg2+^/Q390X^ confirmed the in vivo findings, further describing molecular mechanisms underlying celastrol neuroprotection, i.e., the activation of the AKT-associated pathways, the alteration of the NF-KB-mediate cascade and the synaptic protection, via the induction of anti-inflammatory processes. However, in contrast with the above-mentioned findings, a recent publication showed that celastrol did not impact kindling progression but reduced post-kindling seizure thresholds and enhanced microglia activation in CA1 and CA3 regions of hippocampus [76]. 

## 8. Conclusions

**Figure 2:**
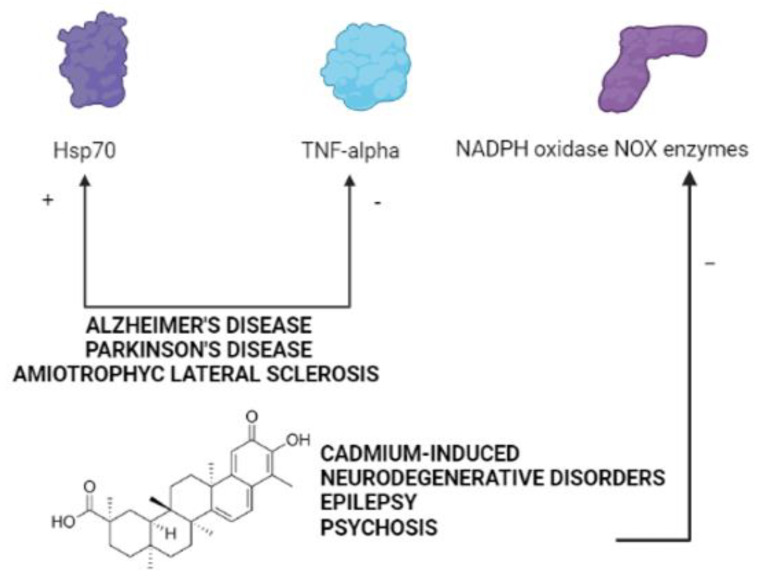
Common molecular targets of celastrol in psychiatric and neurological disorders (created in BioRender.com (accessed on 27 July 2021)).

Although the results described in most of the available in vitro and in vivo studies define celastrol as a promising candidate for the treatment of CNS disorders, no clinical trials to validate this consideration and to finally translate data obtained on cell lines or rodents to humans are available so far. An immediate question which arises from this ascertainment relates to the possible reasons of this lack, especially when considering that clinical trials using celastrol have been largely conducted for other diseases, such as Crohn’s disease, psoriasis, rheumatoid arthritis, diabetes, kidney disorders, and transplantation. One of the possible answer to this issue is certainly related to the need of more consistent preclinical results, especially regarding possible molecular targets of this compound which might be common in many psychiatric and neurological problems (Figure 2) and/or still unknown mechanisms of action and toxic effects before proceeding with clinical trials, as also highlighted by a recent study investigating the effects of celastrol administration in ALS patients [77]. 

Another important aspect which may limit the development of clinical trials for the use of celastrol in CNS disorders is that results obtained from preclinical studies do not refer to the majority of CNS disorders. Indeed, data on other major neuropsychiatric diseases such as anxiety, bipolar disorders, autism, as well as sleep disorders are definitely missing. Therefore, novel experimental proposals in this sense are needed in order to further enrich the still developing scenario of celastrol therapeutic opportunities. 

## Figures and Tables

**Table 1:** Effects of celastrol in Alzheimer’s disease, Parkinson’s disease, and Amiotrophyc lateral sclerosis.

Neurodegenerative Disease	In Vitro/In Vivo	Celastrol Effects	Doses/IC_50_	References
***Alzheimer’s disease***	In vitro - Human monocytes and macrophages - endothelial cells	- Suppression of IL-1βproduction	30 nM	[31]
		- Suppression of TNF-α production	70 nM	
		- Decrease of the production of induced but not constitutive nitric oxide	50 nM IC_50_ = 100 nM	
	In vivo - LPS rat model	- Improvement of memory, learning and performance in psychomotor activity tests	7 µg/kg i.p.	
	In vitro - Stable NF-κB luciferase reporter cell line of HEK293 cells - 7 W CHO cells overexpressing wild-type human APP	- Prevention of NF-κB activation	IC50 < 1 μM	[32]
		- Inhibition of BACE-1 expression	5 μM	
	In vivo *-* Transgenic mice overexpressing the human APP695sw mutation and the presenilin-1 mutation M146L (Tg PS1/APPsw)	- Reduction of APP beta-cleavage with consequently inhibition of Aß_1–40_ and Aß_1–42_ production- Decrease of both soluble and insoluble Aß_1–38_, Aß_1–40_ and Aß_1–42_ levels - Reduction of Aß plaque burden -Microglia activation	2.5 mg/kg/day s.c. long-lasting	
	In vitro - H4 human neuroglioma cells transfected to overexpress human full length APP	- Reduction of Aß production induced by LPS - Increase of Hsp70 and Bcl-2 expression - Decrease of NF-κB activity - Induction of GSK-3β posphorylation at tyrosine 216 - Reduction of COX2 expression - Decrease of Aß accumulation	1, 10, and 100 nM (dose-dependently)	[33]
	In vitro - SH-SY5Y cells treated with Aβ_1-42_	- Inhibition of Tau hyperphosphorylation and Hsp90 expression, induced by Aβ_1–42_ - No effects on the decreased HSP70 and HSF-1 expression, Tau ubiquitination, and HSP70/Tau- HSP70/CHIP interaction induced by Aβ_1–42_	600 nmol/L	[34]
***Parkinson’s disease***	In vitro - Mouse primary cortical neurons and neuroblastoma SH-SY5Y cells incubated with lactacystin	- Absence of neuroprotective effects under conditions of the ubiquitin-proteasome system inhibition	1 µM (co-treatment) 0.01 an 0.1 µM (pre-treatment)	[35]
		- Reduction of cell viability and enhancement of cell death at high concentrations	1 and 2.5 µM	
	In vivo - Lactacystin rat model	- No effects on the decrease of levels of dopamine and its metabolites - Absence of neuroprotective effects on dopaminergic neurons of the substantia nigra	0.3, 1 or 3 mg/kg/1 mL i.p.	
		- Potentiation of the decrease in the levels of dopamine and its metabolites in the lesioned striatum - Acceleration of the total dopamine metabolism - Enhanced oxidative stress - Decrease in the number and/or density of dopaminergic neurons in the substantia nigra	3 mg/kg/1 mL i.p.	
	In vitro - Human dopaminergic neuronal cell line (SH-SY5Y) treated with rotenone	- Protection from cell-injury induced and death induced by rotenone - Prevention of free radical production - Prevention of mitochondria membrane potential - Inhibition of cytochrome c release - Inhibition of Bax/Bcl-2 changes - Inhibition of caspase-9/3 activation - Inhibition of the activation of the p38 mitogen-activated protein kinase	2.5 nM	[36]
	In vivo - MPTP-treated mice	- Attenuation (48%) of the loss of dopaminergic neurons of the substantia nigra - Reduction of dopamine concentration depletion - Induction of Hsp70 expression in dopaminergic neurons - Decrease of TNF-α and NF-κB immunostaining - Reduction of astrogliosis	3 mg/kg i.p.	[25]
	In vivo - Drosophila DJ-1A model	- Neuroprotective effects on dopaminergic neurons	5 and 20 µg	[37]
	In vitro - Dopaminergic neuronal cell line (SH-SY5Y) treated with treated with MPP^+^	- Reduction of the MPP+-induced dopaminergic neuronal death, mitochondrial membrane depolarization, and ATP reduction	0.1–3 μM celastrol (dose dependently)	[38]
	In vivo - MPTP-treated mice	- Suppression of motor symptoms and neurodegeneration in the substantia nigra and striatum - Enhancement of mitophagy in the striatum	3 mg/kg/day i.p. for 3 days	
***Amyotrophic lateral sclerosis***	In vitro -SOD1^G93A^ transfected NSC34 cells	- Attenuation of H_2_O_2_-induced cell death - Decrease of MDA levels -Enhanced GCLC and GST mRNA expressions -Induction of ERK1/2 and Akt	50 nmol/L	[39]
	In vitro *-* Primary motoneuron cultures treated with staurosporin or H_2_O_2_	- Induction of Hsp70 - Absence of neuroprotective effects - Neurotoxic effects and induction of cell death - Induction of the apoptotic cell death cascade	0.3 and 3 μM	[40]
	In vitro - Differentiated neurons	- Neuroprotective effects via induced Hsp70 expression	0.75 μM	[24,41]
	In vivo - G93A SOD1 transgenic mouse model		2 mg/kg and 8 mg/kg p.o.	
	In vivo - G93A SOD1 transgenic mouse model	- Improvement of weight loss and motor performance - Delay of the onset of the disease - Increase (30%) in the neuronal number in the lumbar spinal cord - Decrease of TNF-α, iNOS, CD40, and GFAP immunoreactivity in the lumbar spinal cord - Increase of Hsp70 immunoreactivity in lumbar spinal cord neurons	2 mg/kg and 8 mg/kg p.o.	[27]
***Huntington’s disease***	In vivo 3-nitropropionic acid rat model	- Decrease of the lesion volume in the striatum	3 mg/kg i.p.	[25]
	In vitro - Cell lines expressing mutant polyglutamine protein	- Reduction of the cell killing	0.4, 0.8 and 1.6 μM	[42]
	In vitro - Striatal cell line from the HdhQ111/Q111 knock-in mouse	- Inhibition of mutant huntingtin aggregation - Reverse of the abnormal cellular localization of full-length mutant huntingtin in mutant HdhQ111/Q111 striatal cells	0.25 μM	[26]

**Table 2:** Effects of celastrol in Cerebral Ischemia, Ischemic Stroke, and Traumatic Brain Injury.

Pathological Condition	In Vitro/In Vivo	Celastrol Effects	Doses or IC_50_	References
**Cerebral Ischemia and Ischemic Stroke**	In vitro - Co-cultures of neurons and microglial cells, as well as neuron cultures, with or without 3 h-lasting oxygen glucose deprivation	- Protection against neuronal cell death caused by oxygen glucose deprivation in neuron-microglia co-cultures but not in neuronal cultures - Induction of M2 microglia phenotype development	0.5 and 1 μM	[65]
	In vivo - Murine model of middle cerebral artery occlusion	- Reduction of the infarct volume - Prevention of neuronal death - Protection against cerebral ischemia-induced neurological dysfunction - Improvement of sensorimotor functions - Promotion of M2 microglia polarization	1 mg/kg i.p.	
	In vivo - Murine model of middle cerebral artery occlusion	- Reduction of brain water content - Reduction of p-JNK, p-c-Jun and NF-κB expression	2–3 mg/kg i.p.	[66]
	In vivo *-* Rabbit model of carotid atherosclerosis	- Decrease of both plaque and arterial wall cross-section areas - Decrease of VEGF expression.	1 mg/kg/day and 3.5 mL/kg/day gavage	[67]
**Traumatic brain injury**	In vivo - TBI mouse model	- Improvement of TBI-induced neuronal death and behavioral alterations	1 mg/kg i.p.	[68,69]

## Data Availability

Not Applicable.

## Supplementary Materials

The following are available online. File S1: Combinations of keywords used for literature search.
