# Supplementary material for: The Therapeutic Potential of Celastrol in Central Nervous System Disorders: Highlights from In Vitro and In Vivo Approaches
Source: Molecules. 2021 Aug 3;26(15):4700. doi: 10.3390/molecules26154700 (PMC8347599; doi:10.3390/molecules26154700)
Supplement: Supplementary file 1 [file molecules-26-04700-s001.zip › molecules-1310602-supplementary.pdf]

Combinations of key words used for literature search

Celastrol AND central nervous system; celastrol AND central nervous system disorders; celastrol AND central nervous system diseases; celastrol AND CNS, celastrol AND CNS disorders, celastrol AND CNS diseases; Tripterygium wilfordii AND central nervous system; Tripterygium wilfordii AND central nervous system disorders; Tripterygium wilfordii AND central nervous system diseases; Tripterygium wilfordii AND CNS; Tripterygium wilfordii AND CNS disorders; Tripterygium wilfordii AND CNS diseases; Thunder God Vine AND central nervous system; Thunder God Vine AND central nervous system disorders; Thunder God Vine AND central nervous system diseases; Thunder God Vine AND CNS, Thunder God Vine AND CNS disorders, Thunder God Vine AND CNS diseases; celastrol AND neurodegeneration; celastrol AND neurodegenerative diseases; celastrol AND neurodegenerative disorders; Tripterygium wilfordii AND neurodegeneration; Tripterygium wilfordii AND neurodegenerative disorders; Tripterygium wilfordii AND neurodegenerative diseases; Thunder God Vine AND neurodegeneration; Thunder God Vine AND neurodegenerative diseases; Thunder God Vine AND neurodegenerative disorders; celastrol AND Alzheimer's disease; Tripterygium wilfordii AND Alzheimer's diseases; Thunder God Vine AND Alzheimer's disease; celastrol AND AD; Tripterygium wilfordii AND AD; Thunder God Vine AND AD; celastrol AND beta amyloid; Tripterygium wilfordii AND beta amyloid, Thunder God Vine AND beta amyloid; celastrol AND Aß; Tripterygium wilfordii AND Aß; Thunder God Vine AND Aß; celastrol AND Parkinson's disease; Tripterygium wilfordii AND Parkinson's disease; Thunder God Vine celastrol AND Parkinson's disease; celastrol AND PD; Tripterygium wilfordii AND PD; Thunder God Vine AND PD; celastrol AND Amiotrophyc lateral sclerosis; Tripterygium wilfordii AND Amiotrophyc lateral sclerosis; Thunder God Vine AND Amiotrophyc lateral sclerosis; celastrol AND ALS; Tripterygium wilfordii AND ALS; Thunder God Vine AND ALS; celastrol AND Huntington's disease, celastrol AND HD; Tripterygium wilfordii AND Huntington's disease, Tripterygium wilfordii AND HD; Thunder God Vine AND Huntington's disease; Thunder God Vine AND HD; celastrol AND multiple sclerosis; celastrol AND MS; Tripterygium wilfordii AND

multiple sclerosis; Tripterygium wilfordii AND MS; Thunder God Vine AND multiple sclerosis; Thunder God Vine AND MS; celastrol AND contaminants; celastrol AND toxic contaminants; Tripterygium wilfordii AND contaminants; Tripterygium wilfordii AND toxic contaminants; Thunder God Vine AND contaminants; Thunder God Vine AND toxic contaminants; celastrol AND cadmium; Tripterygium wilfordii AND cadmium; Thunder God Vine AND cadmium; celastrol AND cerebral ischemia; Tripterygium wilfordii AND cerebral ischemia; Thunder God Vine AND cerebral ischemia; celastrol AND stroke; Tripterygium wilfordii AND stroke; Thunder God Vine AND stroke; celastrol AND ischemic stroke; Tripterygium wilfordii AND ischemic stroke; Thunder God Vine AND ischemic stroke; celastrol AND brain injury; Tripterygium wilfordii AND brain injury; Thunder God Vine AND brain injury; celastrol AND traumatic brain injury; Tripterygium wilfordii AND traumatic brain injury; Thunder God Vine AND traumatic brain injury; celastrol AND epilepsy; Tripterygium wilfordii AND epilepsy, Thunder God Vine AND epilepsy; celastrol AND seizures, Tripterygium wilfordii AND seizures; Thunder God Vine AND seizures; celastrol AND neuropsychiatric disorders; Tripterygium wilfordii AND neuropsychiatric disorders; Thunder God Vine AND neuropsychiatric disorders; celastrol AND mood disorders; Tripterygium wilfordii AND mood disorders; Thunder God Vine AND mood disorders; celastrol AND depression; Tripterygium wilfordii AND depression; Thunder God Vine AND depression; celastrol AND major depression; Tripterygium wilfordii AND major depression; Thunder God Vine AND major depression; celastrol AND bipolar disorders; Tripterygium wilfordii AND bipolar disorders; Thunder God Vine AND bipolar disorders; celastrol AND mania; Tripterygium wilfordii AND mania; Thunder God Vine AND mania; celastrol AND hypomania; Tripterygium wilfordii AND hypomania; Thunder God Vine AND hypomania; celastrol AND psychosis; Tripterygium wilfordii AND psychosis; Thunder God Vine AND psychosis; celastrol AND schizophrenia; Tripterygium wilfordii AND schizophrenia; Thunder God Vine AND schizophrenia; celastrol AND anxiety; Tripterygium wilfordii AND anxiety; Thunder God Vine AND anxiety; celastrol AND autism; Tripterygium wilfordii AND autism; Thunder God Vine AND autism; celastrol AND autism spectrum disorders; Tripterygium wilfordii

AND autism spectrum disorders; Thunder God Vine AND autism spectrum disorders; celastrol AND sleep disorders; Tripterygium wilfordii AND sleep disorders; Thunder God Vine AND sleep disorders.

The above-mentioned keyword combinations have been also further combined with the following terms: *in vitro*, *in vivo*, mouse, rats, animal models, cells, cell line, neurons, microglia, macrophages, astrocytes.
